# Familial segmental spinal myoclonus: a rare clinical feature of Friedreich’s ataxia

**DOI:** 10.1186/s40064-015-1121-5

**Published:** 2015-07-08

**Authors:** Rajendra Singh Jain, Sunil Kumar, Shankar Tejwani

**Affiliations:** Department of Neurology, Sawai Man Singh Medical College, Jaipur, Rajasthan India; Department of Radiology, Sawai Man Singh Medical College, Jaipur, Rajasthan India

**Keywords:** Frataxin gene, Atypical phenotype, Friedreich ataxia, Segmental spinal myoclonus

## Abstract

**Introduction:**

Friedreich’s ataxia (FRDA) is the most common autosomal recessive inherited ataxia. It is characterized by onset before the age of 25 year, progressive limb and truncal ataxia, lower limb areflexia, extensor plantars, dysarthria and impaired posterior column sensations. Other important associated features are skeletal deformity, hypertrophic cardiomyopathy and diabetes mellitus. Most of the patients (98%) have an unstable homozygous trinucleotide (GAA) expansion in intron-1 of chromosome 9 and 2% patients are compound heterozygous for GAA expansion and point mutations.

**Case description:**

We observed an adolescence onset FRDA exhibiting spinal segmental myoclonus (SSM) in a family. Triplet repeat primed polymerase chain reaction (TP-PCR) demonstrated unstable expansion of >66 GAA repeats.

**Conclusions:**

SSM is a unique and rare manifestation of FRDA. This might be the first case report of SSM in FRDA patient.

**Electronic supplementary material:**

The online version of this article (doi:10.1186/s40064-015-1121-5) contains supplementary material, which is available to authorized users.

## Background

Friedreich’s ataxia (FRDA) is the most common autosomal recessive inherited ataxia, with onset before 25 year of age. It is characterized by progressive limb and truncal ataxia, lower limb areflexia, extensor plantars, dysarthria and loss of joint position and vibration sensations. Other important associated features are skeletal deformity, hypertrophic cardiomyopathy and diabetes mellitus. FRDA is due to mutation of frataxin gene on the long arm of chromosome 9 (Saccà et al. 2012; Campuzano et al. 1996). Majority of the patients (98%) have an unstable homozygous expansion of GAA trinucleotide whereas, 2% patients are compound heterozygous for GAA expansion and point mutations. GAA repeat expansion length is directly related to the disease severity and inversely related to the age of disease onset (Filla et al. 1996). Point mutation in frataxin gene has been associated with various atypical clinical features (Cossée et al. 1999; McCormack et al. 2000; Zhu et al. 2002). We have observed spinal segmental myoclonus (SSM) with unstable expansion of GAA repeats in frataxin gene.

## Case description

A 19-year-old boy born out of non-consanguineous marriage with normal birth and developmental history, presented with insidious onset, gradually progressive neurologic illness for 9 years. He developed progressive unsteadiness of gait resulting in swaying on either side while walking. His parents also noticed progressive slurring of speech and scoliosis of thoracic spine. He developed involuntary, brief jerky movements of his upper limbs for last 2 years. The severity of illness had increased to such an extent that the patient became bed-bound for 6 months. There was no history of fever, headache, vomiting, tremor, visual impairment, hearing loss, abnormal behavior, cognitive decline, seizures, altered sensorium, bladder symptoms, diabetes mellitus, alcohol or drug abuse.

On physical examination, thoracic scoliosis (convexity toward the left side) and pes cavus deformities were present. Conjunctival telangiectasia and slit-lamp examination for Kayser–Fleischer rings were negative. Higher cognitive functions and cranial nerves examination including bilateral fundi were normal except gaze evoked nystagmus in both the eyes. Motor system examination revealed hypotonia, distal amyotrophy and weakness (Medical Research Council Grade 4/5) in all the four limbs. Interestingly, frequent abnormal, brief, jerky movements were observed in both upper limbs (Additional file 1: Video). These involuntary jerky movement of arms appeared spontaneously without pain or abnormal behavior. It involved the supraspinatus, deltoid, biceps and brachioradialis muscles. It used to worsen by fatigue and mental stress, but not by change in posture or position. The jerks were not observed during sleep. Generalized areflexia and positive bilateral Babinski sign were present. Pain, touch and temperature sensations were intact but vibration and joint position senses were impaired below the knees. Romberg’s sign was positive. Pancerebellar features were present including dysarthria with scanning character, gaze evoked nystagmus with fast component toward the side of gaze, finger–nose–finger and heel-to-shin incoordination, dysdiadochokinesia, truncal and limb ataxia.

**Figure 1:**
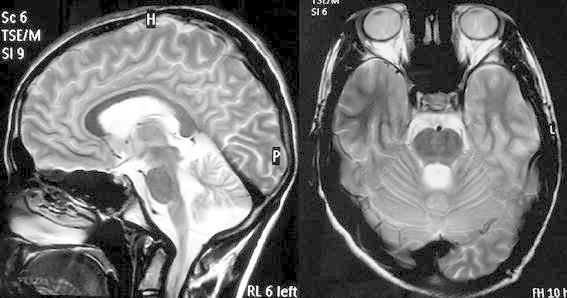
Magnetic resonance imaging (MRI) of brain showing cerebellar atrophy.

Routine hemogram and serum biochemistry including vitamin B12 level and thyroid function tests were normal. Peripheral blood smear for acanthocytes was negative. Electrocardiography (ECG) and 2D-Echocardiography were normal. The compound motor action potentials (CMAPs) showed decreased amplitude in bilateral median, ulnar, peroneal and tibial nerves. The sensory nerve action potentials (SNAPs) were non-recordable in bilateral sural and superficial peroneal nerves. Conventional electroencephalogram (EEG) and 24-h video-EEG did not show any epileptic activity during abnormal movement of limbs. Somatosensory evoked potentials (SSEP) was normal. Surface electromyography (EMG) recordings of supraspinatus, deltoid, biceps and brachioradialis muscles showed semi-rhythmic, myoclonic bursts with a rate of 3–4 Hz without activity in other muscles. The duration of EMG bursts was variable ranging 100–350 ms in these muscles. From surface EMG, the myoclonus seems to originate in the C5–C6 spinal cord segments. We also performed EEG–EMG recording and EEG back-averaging technique to rule out any cortical association. Magnetic resonance imaging (MRI) of spine and brain showed scoliosis of dorso-lumbar region with convexity towards the left side and vermian atrophy, respectively (Figure 1). Genetic study of nucleated cells in peripheral blood by “triplet repeat primed PCR (polymerase chain reaction) Assay” of frataxin gene revealed expansion of >66 GAA repeats in the intron 1.

The patient had four siblings including two sisters and two brothers. One of his 12 years old younger brother had similar complaints of progressive scoliosis, involuntary, brief, jerky movement of arms, unsteadiness of gait and swaying toward either side during walking for 3 years. Genetic counseling was done and prognosis of disease was explained to the family members. He was started on high dose coenzyme-Q and supportive treatment along with physiotherapy and occupational therapy. Clonazepam (0.5 mg twice a day) was given for spinal segmental myoclonus. There was no response to treatment. He is under regular follow up for the last 2 years with worsening of symptoms.

## Discussion and evaluation

Myoclonus is a movement disorder, presenting with sudden, brief, shock-like, involuntary activity of a muscle or group of muscles. It may be either positive results from brief burst of muscle activity or negative due to brief cessation of ongoing muscle contraction. Based on presumed source of generator, myoclonus can be classified as spinal, subcortical or cortical (Shibasaki and Hallett 2005). This physiological classification is the most practical in our clinical practice, since the localization of source guides toward the effective treatment (Caviness and Brown 2004). Babinski first described spinal myoclonus in 1913 (Babinski 1913). It is limited to few contiguous myotomes and may occur irregularly or semi-rhythmic, with the frequency ranges from 1–2 per minute to 100–200 per minute. They are resistant to supraspinal influences like voluntary actions or sleep.

Since spinal cord has propriospinal pathways and segmental organization, spinal myoclonus is further subdivided into propriospinal or segmental myoclonus (Brown et al. 1994). In propriospinal myoclonus, spinal cord generators recruit axial muscles via long propriospinal pathways (Nishiyama et al. 1994). It involves the flexion of axial muscles (neck, trunk and hips). Bursts activity on EMG are usually long-lasting for several hundred milliseconds whereas, spinal segmental myoclonus (SSM) has semi-rhythmic, involuntary contractions of muscle or a group of muscles supplied by a limited contiguous spinal cord segments. They are usually stimulus insensitive and exaggerated by fatigue and emotional stress. This rare type of myoclonus is usually rhythmic and slow (<4 Hz). There are no associated EEG changes.

The diagnosis of spinal myoclonus should include a detailed history and examination with emphasis on the age at onset, exacerbating or relieving factors, progression, family history and associated other neurological symptoms such as ataxia, visual symptoms, epilepsy and cognitive decline. Clinical electrodiagnostic tests (surface electromyography, electroencephalography, combined EEG–EMG study, somatosensory evoked potentials) are important for physiological classification of myoclonus (spinal, subcortical or cortical myoclonus). Surface electromyography recording is useful to determine duration, distribution and stimulus sensitivity of myoclonus. Though, clonazepam and levetiracetam have been tried in spinal myoclonus with variable success, (Keswani et al. 2002) pharmacotherapy is usually unsatisfactory.

Our patient fulfilled the clinical criteria for FRDA: adolescent onset, progressive severe limb and truncal ataxia, dysarthria, nystagmus, generalized areflexia, extensor plantar response, axonal large fiber peripheral neuropathy, distal amyotrophy, scoliosis, pes cavus and positive family history (Harding 1981). Ancillary studies, neurophysiology and MRI brain also supported the diagnosis (Geoffroy et al. 1976). Finally, genetic analysis confirmed the diagnosis of FRDA. Other differential diagnosis like ataxia telangiectasia, spinocerebellar ataxias, hereditary motor and sensory polyneuropathy or alcohol abuse were ruled out by appropriate clinical evaluation and laboratory investigations.

The genetic study of GAA expansion and point mutation for frataxin gene has broadened the clinical spectrum of FRDA. Phenotypic variability are more common in point mutations rather than homozygous expansion of triplet repeats. In our patient, genetic analysis was done by using “triplet repeat primed PCR (TP-PCR)” method, which showed unstable expansion of >66 GAA repeats in the intron 1. TP-PCR based genetic analysis detects only GAA repeats i.e. either homozygous or heterozygous will be positive by TP-PCR method. It is important to rule out point mutations, as most of the phenotypic variability in FRDA has been observed among patients harboring point mutations (Potdar and Raghu 2013; Chattopadhyay et al. 2004). Our patient had financial constraint. Therefore, we could perform TP-PCR based genetic analysis only, which confirmed the diagnosis of FRDA. There are very few case reports described in the literature regarding association of abnormal involuntary movements and Friedreich ataxia. Hanna et al. (1998) reported two patients of FRDA exhibiting generalized chorea who were homozygous for frataxin gene. Zhu et al. (2002) described atypical presentation (chorea and myoclonus) of FRDA due to deletion of frataxin gene. Along with the classic presentation, our patient had a unique and rare clinical feature of SSM.

During myoclonic jerks, our patient was conscious, oriented and following commands and his conventional EEG, video EEG and SSEP were normal, which ruled out the possibility of cortical and subcortical myoclonus. Surface EMG showed semi-rhythmic, myoclonic bursts of 3–4 Hz in C5–C6 innervated myotomes (supraspinatus, deltoid, biceps and brachioradialis). EEG–EMG recording and EEG back-averaging technique were also performed to rule out any cortical association. Similar myoclonus in his younger sibling and development of spinal myoclonus after a long time from the disease onset, clearly ruled out any coincidental occurrence.

Though, the primary abnormality is in the spinal cord, SSM is usually associated with multiple etiologic factors such as infection, myelitis, spinal cord tumors, spinal cord trauma, syringomyelia, cervical spondylosis and vascular malformations. The etiopathogenesis of spinal segmental myoclonus remains unknown. Various postulated mechanisms are re-excitation of local aberrant axons, abnormal hyperactivity of segmental anterior horn cells, local inhibitory loss of dorsal horn interneurons and inhibitory loss from suprasegmental descending pathways (Jankovic and Pardo 1986). We speculate that the possible mechanism of spinal segmental myoclonus in our patient could be related to spinal cord damage due to long-standing disease or may be due to abnormal nucleotide expansion in frataxin gene.

## Conclusions

SSM is a unique and rare manifestation of FRDA. This might be the first case report of SSM in FRDA patient.

## Consent

The patient and his parents gave written consent for the use of personal and medical information for the publication of this case report and any accompanying images.

## Additional file

Additional file 1:
**Video.** Video clip showing sudden, involuntary, brief, jerky movement in both the arms appearing spontaneously without pain or abnormal behavior. It involved the supraspinatus, deltoid, biceps and brachioradialis muscles, but not the other limb muscles. It worsened by mental stress, but not by the posture or position. During episodes patient was conscious and well oriented.

## Abbreviations

MRI: magnetic resonance imaging
CT: computed tomography
TP-PCR: triplet repeat primed PCR
SSM: spinal segmental myoclonus
FRDA: Friedreich’s ataxia

## References

[CR1] Babinski J (1913). Contracture liée à une irritation des cornes antérieures de la moelle dans un cas de syringomyélie. Rev Neurol.

[CR2] Brown P, Rothwell JC, Thompson PD, Marsden CD (1994). Propriospinal myoclonus: evidence for spinal “pattern” generators in humans. Mov Disord.

[CR3] Campuzano V, Montermini L, Moltò MD, Pianese L, Cossée M, Cavalcanti F (1996). Friedreich’s ataxia: autosomal recessive disease caused by an intronic GAA triplet repeat expansion. Science.

[CR4] Caviness JN, Brown P (2004). Myoclonus: current concepts and recent advances. Lancet Neurol.

[CR5] Chattopadhyay B, Gupta S, Gangopadhyay PK, Das SK, Roy T, Mukherjee SC (2004). Molecular analysis of GAA repeats and four linked bi-allelic markers in and around the frataxin gene in patients and normal populations from India. Ann Hum Genet.

[CR6] Cossée M, Dürr A, Schmitt M, Dahl N, Trouillas P, Allinson P (1999). Friedreich’s ataxia: point mutations and clinical presentation of compound heterozygotes. Ann Neurol.

[CR7] Filla A, De Michele G, Cavalcanti F, Pianese L, Monticelli A, Campanella G (1996). The relationship between trinucleotide (GAA) repeat length and clinical features in Friedreich ataxia. Am J Hum Genet.

[CR8] Geoffroy G, Barbeau A, Breton G, Lemieux B, Aube M, Leger C (1976). Clinical description and roentgenologic evaluation of patients with Friedreich’s ataxia. Le J Can Des Sci Neurol.

[CR9] Hanna MG, Davis MB, Sweeney MG, Noursadeghi M, Ellis CJ, Elliot P (1998). Generalized chorea in two patients harboring the Friedreich’s ataxia gene trinucleotide repeat expansion. Mov Disord.

[CR10] Harding AE (1981). Friedreich’s ataxia: a clinical and genetic study of 90 families with an analysis of early diagnostic criteria and intrafamilial clustering of clinical features. Brain.

[CR11] Jankovic J, Pardo R (1986). Segmental myoclonus. Arch Neurol.

[CR12] Keswani SC, Kossoff EH, Krauss GL, Hagerty C (2002). Amelioration of spinal myoclonus with levetiracetam. J Neurol Neurosurg Psychiatry.

[CR13] McCormack ML, Guttmann RP, Schumann M, Farmer JM, Stolle CA, Campuzano V (2000). Frataxin point mutations in two patients with Friedreich’s ataxia and unusual clinical features. J Neurol Neurosurg Psychiatry.

[CR14] Nishiyama K, Ugawa Y, Takeda K, Sakuta M (1994). Axial myoclonus mediated by the propriospinal tract: a case report. Eur Neurol.

[CR15] Potdar PD, Raghu A (2013). Molecular diagnosis of Friedreich ataxia using analysis of GAA repeats and FXN gene exons in population from Western India. Asian J Neurosci.

[CR16] Saccà F, Marsili A, Puorro G, Antenora A, Pane C, Tessa A (2012). Clinical use of frataxin measurement in a patient with a novel deletion in the FXN gene. J Neurol.

[CR17] Shibasaki H, Hallett M (2005). Electrophysiological studies of myoclonus. Muscle Nerve.

[CR18] Zhu D, Burke C, Leslie A, Nicholson GA (2002). Friedreich’s ataxia with chorea and myoclonus caused by a compound heterozygosity for a novel deletion and the trinucleotide GAA expansion. Mov Disord.

